# Challenges of pheromone-based mating disruption of *Cydia strobilella* and *Dioryctria abietella* in spruce seed orchards

**DOI:** 10.1007/s10340-017-0929-x

**Published:** 2017-11-07

**Authors:** Glenn P. Svensson, Hong-Lei Wang, Erling V. Jirle, Olle Rosenberg, Ilme Liblikas, J. Michael Chong, Christer Löfstedt, Olle Anderbrant

**Affiliations:** 1 Department of Biology, Lund University, 223 62 Lund, Sweden; 2 The Forestry Research Institute of Sweden, Skogforsk, Uppsala, Sweden; 3 Institute of Technology, University of Tartu, Tartu, Estonia; 4 Department of Chemistry, University of Waterloo, Waterloo, Canada

**Keywords:** Pest management, Lepidoptera, *Picea*, Cone damage, SPLAT

## Abstract

Seed orchards function as the primary source of high-quality seeds for reforestation in many European countries, but their seed yields can be severely reduced due to seed- and cone-feeding insects. We evaluated various parameters of pheromone-based mating disruption for control of the moths *Cydia strobilella* and *Dioryctria abietella*, which are major pests in European *Picea abies* seed orchards. We applied different types of pheromone dispensers (rubber septa or wax droplets) at different densities and heights, and with different amounts of active components, covering whole orchards or part of an orchard. The efficacy of the treatment was evaluated by analysing male captures in pheromone-baited assessment traps, and presence of larvae in cones. A dramatic decrease (94–100%) in capture of males in traps occurred in all pheromone-treated plots compared to control plots for both moth species. In contrast, a subsequent reduction in larval numbers in cones was only achieved when wax droplets were used as the dispensing formulation at high density and at the highest pheromone dose tested, and only in half of the trials for each pest species. Electrophysiological recordings using antennae of male *C. strobilella* indicated elevated pheromone concentrations in a treated plot versus a control plot. Our results show that mating disruption has potential to reduce cone damage in spruce seed orchards caused by *C. strobilella* and *D. abietella*, but optimisation of the technique is required to achieve consistent and efficient population suppression of these pests.

**Electronic supplementary material:**

The online version of this article (10.1007/s10340-017-0929-x) contains supplementary material, which is available to authorized users.

## Keymessage

- The mating behaviour of pest insects can potentially be disrupted by releasing high doses of their sex pheromones.
- In mating disruption trials of the spruce cone pests *Cydia strobilella* and *Dioryctria abietella*, such treatment drastically reduced catches of males in pheromone traps of both species.
- Larval presence and abundance in cones were, however, generally not affected by the treatment.
- Optimisation of the mating disruption method to control the pests is needed to increase the seed yield in spruce seed orchards.

## Introduction

In Europe, coniferous seed orchards are the main source for production of high-quality seeds for reforestation (Ruotsalainen 2014). Trees derived from such seeds are phenotypically superior to trees originating from seeds collected in natural forests. In seed orchards of Norway spruce *Picea abies* (L.) H. Karst, however, the seed yields are too low and irregular to meet the demands, and an important reason for the insufficient seed supply is damage caused by cone-feeding insects (Turgeon et al. 1994; Seifert et al. 2000). The spruce seed moth, *Cydia strobilella* L. (Tortricidae), and the spruce coneworm, *Dioryctria abietella* Denis & Schiffermüller (Pyralidae), are severe pests in these seed orchards, and the proportion of cones infested by these species can reach 80–90% (Seifert et al. 2000; Rosenberg and Weslien 2005; Rosenberg et al. 2015). Although the two species utilise spruce cones as food source, they differ in many life history traits. Females of the diurnal *C. strobilella* oviposit into female spruce flowers, and larvae mainly feed on seeds and remain inside the cone until adult emergence. In contrast, females of the nocturnal *D. abietella* lay eggs on young cones and larvae mainly feed on cone tissue. The last instars exit the cone and drop to the ground, and adults emerge from the soil. The two moths also differ in other features such as flight phenology (Annila 1979, 1981).

Due to their large impact on spruce seed yield, efficient control strategies for *C. strobilella* and *D. abietella* are highly desirable. Damage caused by *D. abietella* can be reduced by treating trees with *Bacillus thuringiensis* var. *kurstaki* x *aizawai* GC-91 (Bt) (Rosenberg and Weslien 2005), or alpha-cypermethrin (Rosenberg et al. 2015). Because *C. strobiella* stays inside the cone until adult emergence, externally applied insecticides are not efficient to control this species. Interference with the olfactory-based mate-finding behaviour in moth pests by applying high doses of synthetic sex pheromones (mating disruption) has become an efficient control method in many crop systems. This strategy has been successfully used to manage several important moth pests in, e.g. vineyards and fruit orchards (Witzgall et al. 2010), which are perennial cropping systems with habitats similar to spruce seed orchards with respect to size and degree of spatial isolation. The female-produced sex pheromones of *C. strobilella* and *D. abietella* have recently been identified (Wang et al. 2010; Löfstedt et al. 2012), and efficient pheromone-baited traps are now used for population monitoring of these pests in Sweden, other Nordic countries, and Estonia (Rosenberg et al. 2010). Although mating disruption has worked well for control of many lepidopteran pests in agriculture and forestry, not all attempts to use this technique have been successful (Witzgall et al. 2010; Cardé and Minks 1995). The lack of efficient mating disruption can be due to an insufficient number of pheromone point sources or a low release rate of pheromone. A high density of the target species, immigration of mated females into the treated area, habitat features, weather conditions, as well as a number of physiological and behavioural mechanisms are other factors potentially influencing the efficiency of the pheromone treatment (see Miller and Gut (2015) for a comprehensive review).

Various technologies have been developed over the years to optimise the release of pheromone for efficient mating disruption, including hand-applied reservoir-type dispensers, sprayable dispensers, machine-applied formulations, and controlled release devices (Miller and Gut 2015). Specialised Pheromone and Lure Application Technology (SPLAT) represents a recently developed technique using a wax matrix formulation in which the behaviourally active compounds are incorporated and applied to the vegetation (Stelinski et al. 2005). Compared to traditional hand-applied dispensers, SPLAT dispensers are inexpensive to produce and the formulation is biodegradable and provides long-term rain and UV protection for the active ingredients. Droplet size and shape can be varied, making the system flexible for application of point sources and release rate of pheromone over time in the cropping area. The technique has been used successfully to suppress mating of, e.g. *Paralobesia viteana* (Clemens) and *Epiphyas postvittana* (Walker) in vineyards (Jenkins and Isaacs 2008; Suckling et al. 2012), *Grapholita molesta* (Busck) in apple orchards (Stelinski et al. 2007), and *Phyllocnistis citrella* Stainton in citrus orchards (Lapointe et al. 2014).

Previous attempts to use mating disruption for control of pest insects in spruce seed orchards have been restricted to Nearctic species. *Cydia youngana* Kearfott, which was previously considered taxonomically synonymous with *C. strobilella* (Grant et al. 1989; Bédard et al. 2002), but now found to be genetically and pheromonally distinct to its European sister species (Svensson et al. 2013), is a serious pest in seed orchards of white spruce, *Picea glauca* (Moench) Voss. In experiments in which one 2.5 ha plot in each of two seed orchards was treated with high doses of pheromone, trap catch of males was reduced by up to 98% in treated areas versus control areas, and the proportion of infested cones was reduced by up to 69% in a treated plot versus a control plot (Trudel et al. 2006). In experiments performed in a seed orchard of loblolly pine, *Pinus taeda* L., where 1.2 ha was pheromone-treated, trap catch of *Dioryctria amatella* (Hulst), *Dioryctria disclusa* (Heinrich), and *Dioryctria merkeli* (Mutuura and Munroe) was reduced by 91, 99, and 97%, respectively (DeBarr et al. 2000). Cone damage caused by these moths, however, was not analysed, making it difficult to evaluate the efficacy of the pheromone treatment.

The objective of this study was to test whether mating disruption has the potential to control *C. strobilella* and *D. abietella* in European spruce seed orchards. We have identified the sex pheromone of *C. strobilella* as a blend of (8*E*,10*E*)-dodecadienyl acetate (*E*8,*E*10-12:OAc) and (8*E*,10*Z*)-dodecadienyl acetate (*E*8,*Z*10-12:OAc), produced in very small amounts (1 pg per female) (Wang et al. 2010). We have also characterised the sex pheromone of *D. abietella* as a blend of (3*Z*,6*Z*,9*Z*,12*Z*,15*Z*)-pentacosapentaene (C25 pentaene) and (9*Z*,11*E*)-tetradecadienyl acetate (*Z*9,*E*11-14:OAc) (Löfstedt et al. 1983, 2012). In the current study, we conducted experiments during four field seasons, using different dispensing technologies (rubber septa or SPLAT), different densities and heights of pheromone point sources, and different sizes of the treated area in an attempt to demonstrate the disruption effect on the target species. In addition, we performed recordings of electroantennograms (EAG) with antennae of *C. strobilella* males to monitor concentrations of the sex pheromone in a treated area and a control area. Experiments were performed in the few available managed spruce seed orchards in southern and mid-Sweden. The irregular flowering and cone production in these orchards resulted in poor replication in most years of the study, and cone collection could not always be conducted at a certain site due to scarcity of cones. Being aware of these limitations, our study is the first to evaluate mating disruption in European spruce seed orchards.

## Materials and methods

### Chemicals, traps, and baits

Substances of different origin and purity (determined by gas chromatography–mass spectrometry) were used in these experiments, as listed in Table S1. Descriptions of the syntheses of *E*8,*Z*10-12:OAc and C25 pentaene are provided in Appendices 1 and 2, respectively. Transparent plastic delta traps with sticky inserts, purchased from CSalomon (Plant Protection Institute, Hungarian Academy of Science, Budapest, Hungary), were used. Red rubber septa (11 × 5 mm, #224100–020) from Wheaton Science Products (Millville, NJ, USA) were used as lures in traps for experiments on both moth species. Compound solutions were prepared in n-hexane (> 99%, Merck, Darmstadt, Germany) or n-heptane (> 99%, VWR, Fontenay-sous-Bois, France), and baits were produced by applying 100 µl of a solution on the septum. Baits for *C. strobilella* monitoring traps were loaded with 0.15 µg each of *E*8,*E*10-12:OAc and *E*8,*Z*10-12:OAc (amounts of stock solution adjusted for differences in isomeric purity of compounds), whereas baits for *D. abietella* assessment traps were loaded with 0.1 mg of *Z*9,*E*11-14:OAc and 10 mg of C25 pentaene.

### Disruption dispensing systems

Wheaton septa were used as disruption dispensers in experiments conducted in 2010–2012. Septa were pierced with a thin metal wire and hung on spruce branches. In the pilot study targeting *D. abietella* in 2010, release of the C25 pentaene was achieved by applying the compound on a cotton roll inserted into a 1.5-ml glass vial. In experiments conducted in 2015, the SPLAT wax-based formulation was used to dispense pheromone directly onto spruce branches (Stelinski et al. 2005). SPLAT with the pheromone components incorporated into the matrix was supplied by ISCA Technologies (Riverside, CA, USA).

### General design of experiments

Figure S1 and Table S2 provide information about the geographic distribution and size of the sites used, and Table S3 shows the main features of the experiments performed in different years. The sites included six spruce seed orchards for large-scale production of seeds, and one spruce clone archive (Maltesholm). In most experiments, the number of disruption dispensers applied per tree in a treatment plot was adjusted to the number of trees present in such plot to achieve a certain density of point sources. In these experiments, six monitoring traps were applied in the centre of a treatment or control area. Traps were positioned in a circle with a radius of 20 m and with half of the traps being placed at 2 m height and the other half at 4–5 m height. In the pilot study with *D. abietella* in 2010, however, dispensers and traps were applied in a different way (see below). Captures per assessment trap were pooled to give the total number of males trapped per plot to be used in the statistical analysis. Due to poor replication in most years, reliable statistical analysis of catch data could only be done for experiments performed in 2012, when three sites with a treatment plot and a control plot were included. We used a generalised linear model (IBM SPSS statistics v19, New York, NY, USA) to compare the catch in treatment plots versus control plots. Because catch data were overdispersed, we used a negative binomial distribution with log link function as model. To estimate the percentage reduction in trap catch (disruption effect), we used the formula:

$$ {\text{Disruption effect }} = \left( {1{-}\left( {{\text{catch in treatment}}/{\text{catch in control}}} \right)} \right)\; \times \;100 $$

### Electrophysiology

In 2010, a portable electroantennogram sensor, model PortEAG-3 (Syntech, Kirchzarten, Germany) (Van der Pers and Minks 1998), was used to compare relative aerial concentrations of the *C. strobilella* sex pheromone in the mating disruption plot versus the control plot during the experiments in Maltesholm. Male moths, which had emerged from field-collected spruce cones in the laboratory (see Wang et al. (2010) for rearing protocol), or had been collected directly from traps in the control plot at the field site, were used for these experiments. The head with both antennae was mounted in the airtight holder, separating it from the ambient air. The head and antennal tips were covered with electrically conductive gel (Cefar, Lund, Sweden). A Wheaton rubber septum loaded with a 100-µl solution including 1 ng each of *E*8,*E*10-12:OAc and *E*8,*Z*10-12:OAc was used as a reference stimulus during recordings. Between the experiments, the septum was kept in the freezer. A continuous flow of air (0.5 ms^−1^), cleaned through an activated charcoal filter, maintained the non-stimulated baseline condition of the antennal preparation.

Each measurement consisted of a reference recording (R_1_), followed by an ambient air recording (A), and a second reference recording (R_2_). The duration of each recording was 0.3 s, and the time between recordings was set to 5 s. Normalised EAG responses were calculated as:

$$ {\text{Normalised EAG}} = (A/((R_{1} + R_{2} )/2))\; \times \;100 $$

After the experiments, data were transferred to a PC via a serial (RS232) connection and further processed by the Syntech Autospike 32 program (Syntech, Kirchzarten, Germany). EAG measurements were taken during two periods of the experiment: five and seven days after the disruption dispensers had been placed in the treatment plot and six days after they had been removed. For each antennal preparation, recordings were performed at five positions within each plot, both at 2 and 4 m height, generating a mean EAG value for each of these four positions. The time period between recordings was at least 1 min. Whether measurements for a given antennal preparation started in the control or in the treatment plot was randomised. Five antennal preparations were used in each of the two experiments. Differences in the mean normalised EAG values between recording positions were analysed using repeated-measures ANOVA with recording position as independent variable and EAG response as dependent variable (IBM SPSS v19).

### Cone analysis

To evaluate the effect of mating disruption on larval feeding damage caused by the two pests, spruce cones were collected and analysed for presence and abundance of larvae. In the experiments performed in 2011 and 2015, cones were collected in July–August. In 2011, ten spruce trees in each location were randomly selected, and the outermost cone and innermost cone on a branch were collected from five randomly selected branches per tree, giving a total of 100 cones per site. In 2015, the same protocol was used for the treatment plot and the control plot at a given site. For some of the experiments, fewer trees were sampled due to poor cone production. In 2012, an alternative protocol was used. In November, six spruce trees were selected in the first, middle, and last row of the seed orchard with a total of 14 rows in both the MD area and control area. From each tree, all cones on three branches were removed, and from these, three cones were randomly selected for analysis, giving a total of 18 cones per treatment. Each cone was dissected and the presence of larvae and the number of larvae were scored for each moth species. The number of larvae for all cones analysed was pooled to give the total number of larvae per plot. Because the number of cones analysed was sometimes lower than 100, the total number of larvae was divided by the number of cones analysed. The proportion of infested cones and the mean number of larvae per cone per plot were then compared between pheromone-treated plots and control plots.

### Experiments 2010

*Cydia strobilella*: These initial field tests were conducted in Maltesholm and Ålbrunna to investigate if treatment with disruption dispensers resulted in a reduction in trap catch compared to non-treated control areas and if such effect disappeared when dispensers were removed. At each location, 1 ha (100 m × 100 m) was treated with pheromone and the treated area and the control area were separated by at least 200 m. In the treated area, rubber septa loaded with 1 mg each of *E*8,*E*10-12:OAc and *E*8,*Z*10-12:OAc (compensated for differences in isomeric purity of compounds) were placed in an 8 × 8 matrix, giving a density of 64 point sources per ha (similar to the density used by Trudel et al. 2006). Two different mating disruption regimes were tested, suspending the dispensers at 2 m or 4 m height. In Maltesholm, traps were placed in the field 19 May to monitor the abundance of moths in the areas to be used as treatment and control. Disruption dispensers were placed at 4 m height 21 May and present for 1 week. The dispensers were then removed and absent for a week, before being placed at 2 m height and present until the experiment was terminated 10 June. Traps were checked every 2–4 days. In Ålbrunna, the reverse order of treatment was used. Traps were placed in the field 19 May, and disruption dispensers were present at 2 m height 24–28 May, absent 28 May–4 June, and present at 4 m height 4–11 June, when the experiment was terminated. Traps were checked every 2–4 days. In addition, EAG measurements were taken in Maltesholm (see above).

*Dioryctria abietella:* Small-scale field tests were performed in Gringelstad, Hosaby, and Maglehem (one replicate per site) to evaluate the efficacy of different pheromone treatments in reducing trap catches of *D. abietella* males. Three treatments were tested: (i) rubber septum loaded with 100 mg of *Z*9,*E*11-14:OAc, (ii) rubber septum loaded with 100 mg of *Z*9,*E*11-14:OAc together with cotton roll dispenser loaded with 1 g of C25 pentaene, and (iii) cotton roll dispenser loaded with 1 g of C25 pentaene. In each experiment, six dispensers were placed in a circle around a focal tree with a radius of 5 m and at 3–4 m height. As control, a tree without dispensers was used. The distance between focal trees was at least 100 m. Two assessment traps were placed in the focal trees of both pheromone-treated plots and control plots, at 2 m and at 3–4 m height. The tests started 24 June and ended 9 September, and the same dispensers were used throughout the experiments.

### Experiments 2011

*Cydia strobilella* and *D. abietella:* The entire seed orchards in Maglehem and Gälltofta (5.9 ha each) were pheromone-treated for both species, whereas the locations at Maltesholm, Högseröd, Gringelstad, and Hosaby were left untreated. In the treated orchards, the same dispenser type and dispenser density as in the *C. strobilella* experiments 2010 were used. For both species, disruption dispensers were placed at 2 m height. For *C. strobilella*, the same amounts of acetates were used in dispensers as the previous year. Tests were conducted 26 April–23 June, and disruption dispensers were replaced after three weeks. For *D. abietella*, only *Z*9,*E*11-14:OAc was used in disruption dispensers (100 mg per septum) because C25 pentaene has no disruption effect (see below). Experiments were conducted 19 May–27 September, and the same dispensers were used throughout the trials. Traps were checked weekly for both species. Cones were collected from all six locations included in the experiments, using the standard protocol described above, and analysed for larval presence and abundance of both species.

### Experiments 2012

*Cydia strobilella:* Experiments were performed in Maglehem (1 May–20 June), Hosaby (2 May–20 June), and Ålbrunna (8 May–14 June). At each site, 1 ha (100 m × 100 m) was treated with pheromone, and the treatment area and control area were separated by at least 200 m. The same 8 × 8 matrix of dispensers as in 2010 was used, but septa were loaded with a ten times higher dose (10 mg) of each acetate isomer compared to previous tests. All disruption dispensers this year were placed at tree crown level (4–5 m height). Dispensers were replaced after three weeks. Traps were checked weekly. Due to poor spruce flowering at the southern sites, cone collection was only performed at Ålbrunna, and the alternative protocol for collection (mentioned above) was used.

### Experiments 2015

*Cydia strobilella* and *D. abietella:* Experiments were performed in Maltesholm (4 May–26 June) and Hosaby (6 May–26 June) to control *C. strobilella* and in Gringelstad (12 June–1 October) and Maglehem (13 June–1 October) to control *D. abietella*. For each location, 2 ha (200 m × 100 m) was treated with SPLAT at a density of 320 release sources per ha. SPLAT was hand applied using a wooden stick applicator as ≈ 2 g point sources at the upper part of spruce trees accessed by ladder. The formulation for *C. strobilella* included a 3:2 ratio of *E*8,*E*10-12:OAc and *E*8,*Z*10-12:OAc (compensated for differences in isomeric purity of compounds) at a concentration of 20 mg per droplet, resulting in a total dose of 6.4 g per ha, i.e. five times higher dose compared to the tests 2012. A second application of SPLAT was performed after three weeks. The formulation for *D. abietella* included *Z*9,*E*11-14:OAc at a concentration corresponding to 50 g per ha, i.e. ≈ 8 times higher dose compared to the tests in 2011, and a single application was performed. Because the number of spruce trees per ha differed greatly among the sites (Maglehem: 199 trees, Maltesholm: 1050 trees, Gringelstad: 250 trees, Hosaby: 216 trees), the number of point sources per tree was adjusted to get the same total dose of the disruptant per ha for each experiment. At each location, the control area was at least 150 m away from the treated area. Traps were checked weekly for both species. Cones were collected from all four locations, using the standard protocol described above, and analysed for larval presence and abundance of both species.

## Results

### General pattern of trap catches

In all experiments for both moth species, catches of males in control plots were lower in traps placed at 2 m height compared to those placed at 4–5 m height. For *C. strobilella*, high traps caught on average four times as many males compared to low traps, and for *D. abietella*, the difference was even more pronounced with 14 times as many males caught in high traps compared to low traps.

### Experiments 2010

**Fig. 1:**
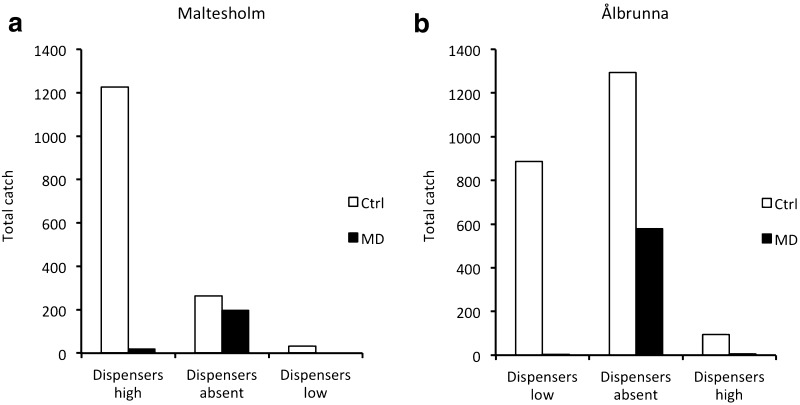
Captures of male *Cydia strobilella* in pheromone-baited traps in a control plot (Ctrl) and a plot with rubber disruption dispensers (MD) in **a** Maltesholm and **b** Ålbrunna in 2010. Disruption dispensers were initially present at either 4 m (high) or 2 m (low) height for about 1 week, then absent for 1 week, and finally present at the alternative height for 1 week

*Cydia strobilella:* After the disruption dispensers had been applied at 4 m height in Maltesholm, a drastic reduction in male catches was observed in the treatment area versus the control area, which corresponded to a disruption effect of 99% (Fig. 1a). Removal of disruption dispensers increased catches in the treatment plot to the same level as in the control plot (Fig. 1a). When dispensers were placed at 2 m height, the disruption effect was 100% (Fig. 1a).

In Ålbrunna, when dispensers initially were placed at 2 m height, the reduction in male catches in the treatment plot versus the control plot corresponded to a disruption effect of 100% (Fig. 1b). This effect largely disappeared after the disruption dispensers had been removed, but when dispensers were placed at 4 m height, catches were again higher in the control plot versus treatment plot, and corresponded to a disruption effect of 94% (Fig. 1b).

**Fig. 2:**
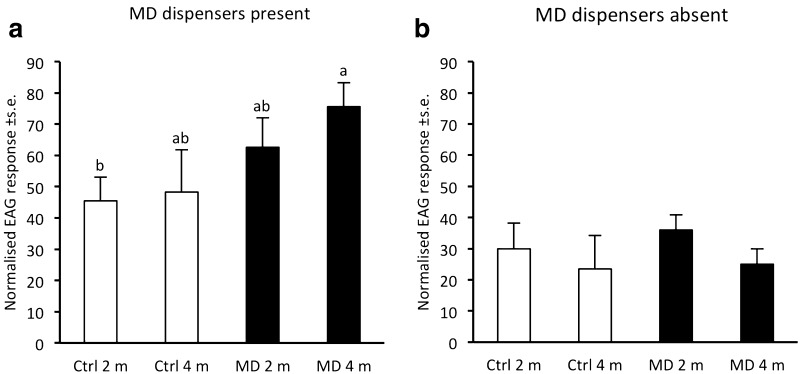
Electroantennogram (EAG) recordings from antennae of male *Cydia strobilella* in a control plot (Ctrl) and a pheromone-treated plot (MD) in Maltesholm in 2010. In each plot, recordings were performed at 2 and 4 m height and experiments were performed when rubber disruption dispensers were present at 4 m height (**a**), and 6 days after dispensers had been removed (**b**). Five antennae were used in each experiment. Bars with different letters indicate significantly different responses (repeated-measures ANOVA; *P* < 0.05)

In the first EAG analysis performed in Maltesholm, with disruption dispensers present, the normalised EAG response was significantly higher for recordings at 4 m height in the treatment plot versus recordings at 2 m in the control plot (*p* = 0.03; Fig. 2a). In contrast, no significant difference in EAG responses was detected between recording positions when measurements were taken 6 days after the dispensers had been removed (Fig. 2b).

In the small-scale study on *D. abietella*, trap catch was reduced to almost zero in treatments including *Z*9,*E*11-14:OAc, whereas trap catch in trees with only C25 pentaene dispensers was the same as catch in the control, showing that only *Z*9,*E*11-14:OAc has a disruptive effect on the species (data not shown).

### Experiments 2011

**Fig. 3:**
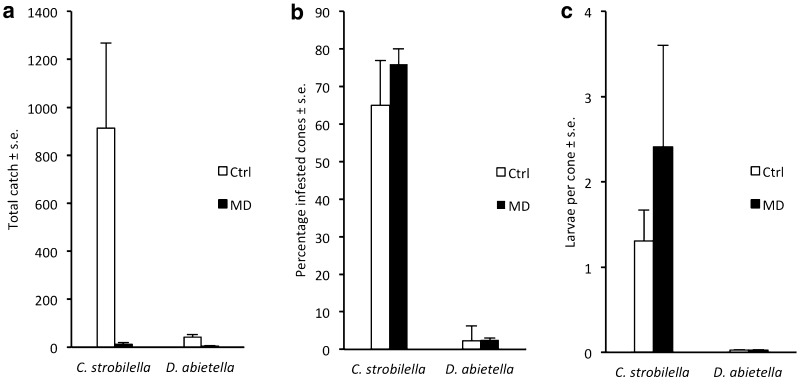
Captures of male *Cydia strobilella* and *Dioryctria abietella* in pheromone-baited traps in two spruce seed orchards with rubber disruption dispensers placed at 2 m height (MD) and four control sites (Ctrl) in 2011 (**a**), and subsequent infestation of cones by these species. Infestation was measured as the frequency of infested cones (**b**) and the number of larvae per cone (**c**)

The seed orchards in Gälltofta and Maglehem, which experienced pheromone treatment, had lower male catches of both species, compared to the remaining sites functioning as “controls” (Fig. 3a). However, the low catches of *C. strobilella* observed in the treated orchards were not correlated with low infestation rates (Fig. 3b, c). In fact, the number of larvae per cone in Gälltofta was at least twice as high as compared to any other site. In addition, evaluating the effect of the pheromone treatment on *D. abietella* mating behaviour was hampered by the fact that four sites (Maglehem, Gälltofta, Gringelstad, and Hosaby) were treated with Bt this year, resulting in a drastic decrease in larval density at all sites compared to 2010 (Fig. 3b, c).

### Experiments 2012

**Fig. 4:**
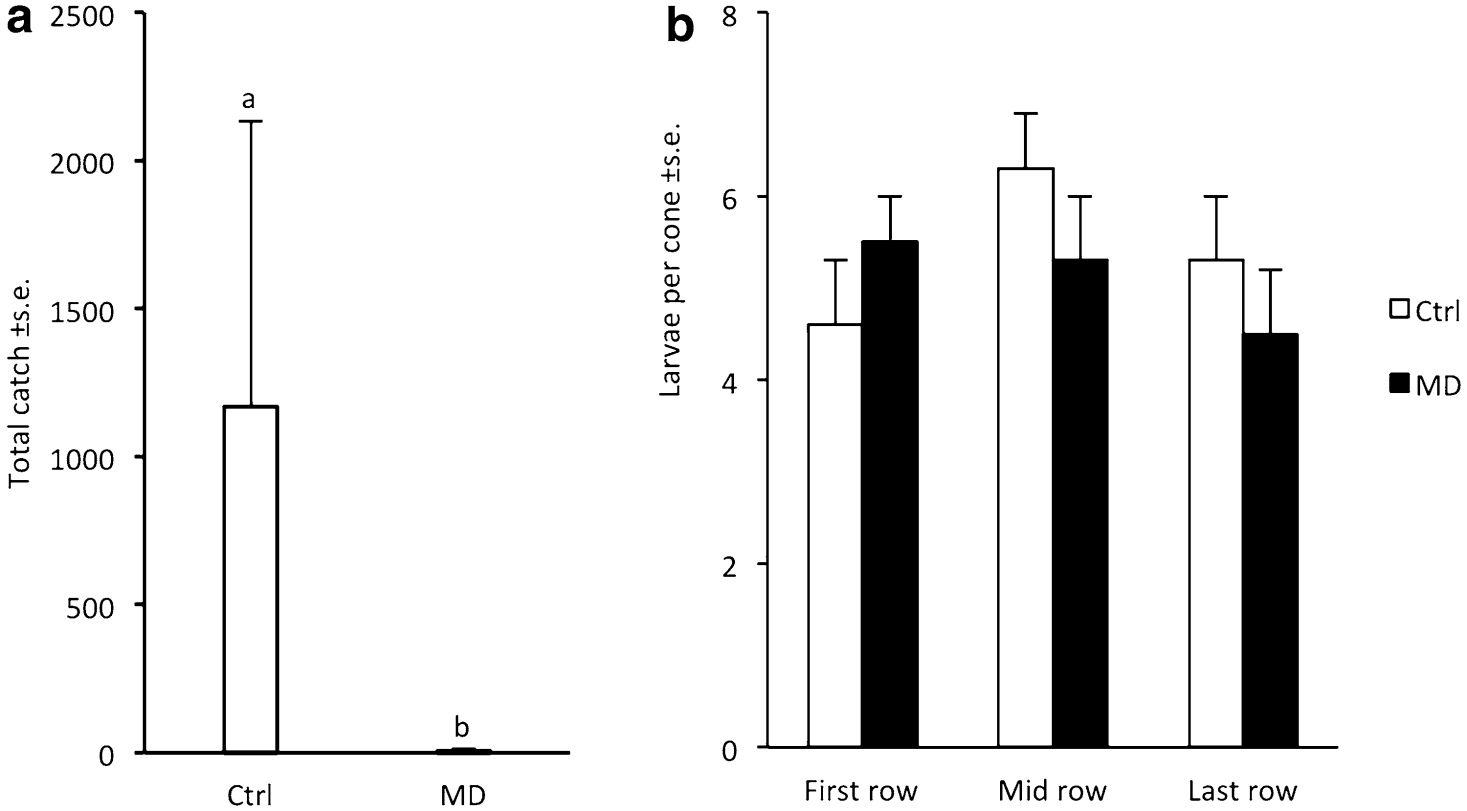
Captures of male *Cydia strobilella* in pheromone-baited traps in control plots (Ctrl) and plots with rubber disruption dispensers placed at 4–5 m height (MD) in three spruce seed orchards in 2012 (**a**) and subsequent infestation of cones by the species in Ålbrunna (**b**). Bars with different letters indicate significant differences in catch data between control and treatment (generalised linear model; *P* < 0.001)

*Cydia strobilella:* Catches of males in pheromone-treated plots were reduced by 99.5 ± 0.3% compared to catches in control plots (*χ* ^*2*^ = 26.9; *d.f.* = 1; *p* < 0.001; Fig. 4a). The analysis of cones collected in Ålbrunna revealed that *C. strobilella* was present in all cones except one, and the number of larvae observed per cone was similar in the treated and untreated area (Fig. 4b).

### Experiments 2015

**Fig. 5:**
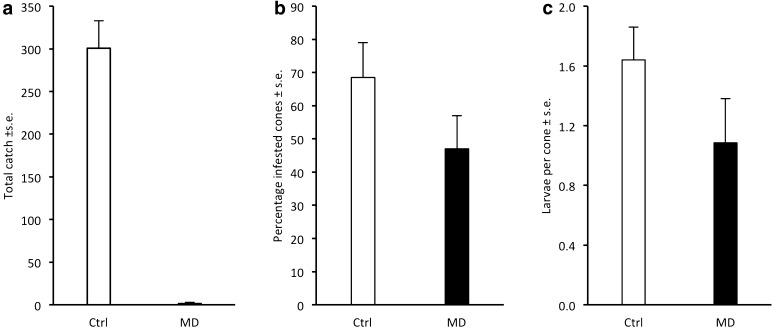
Captures of male *Cydia strobilella* in pheromone-baited traps in control plots (Ctrl) and plots with SPLAT droplets applied at 4–5 m height (MD) at two sites in 2015 (**a**) and subsequent infestation of cones by the species (**b**, **c**)

*Cydia strobilella:* At both sites, the SPLAT treatment suppressed male catches, corresponding to a disruption effect of 99.4 ± 0.6% (Fig. 5a). In Maltesholm, the frequency of infested cones was reduced by 53% in the treatment plot versus the control plot, and the number of larvae per cone was reduced by 58% (Fig. 5b, c). In contrast, no effect of the SPLAT treatment on infestation by *C. strobilella* was observed in Hosaby.

**Fig. 6:**
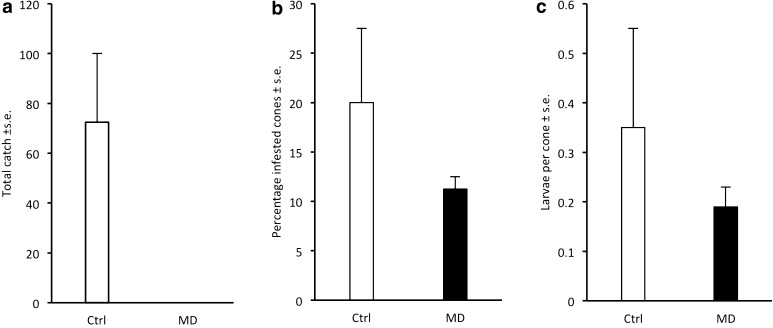
Captures of male *Dioryctria abietella* in pheromone-baited traps in control plots (Ctrl) and plots with SPLAT droplets applied at 4–5 m height (MD) at two sites in 2015 (**a**) and subsequent infestation of cones by the species (**b**, **c**)

*Dioryctria abietella:* No males were captured in traps in the treatment area of the two experimental sites, whereas the average catch in the control plot was above 70 males (Fig. 6a). In Maglehem, the proportion of infested cones was reduced by 64% in the treatment plot versus control plot, and the number of larvae per cone was reduced by 73% (Fig. 6b, c). In Gringelstad, however, the frequency of infested cones and the number of larvae per cone was similar between treatment and control (Fig. 6b, c).

## Discussion

Our study demonstrates the potential to use pheromone-based mating disruption to reduce infestation by *C. strobilella* and *D. abietella* in *P. abies* seed orchards. In all comparative experiments for both species, nearly complete (94–100%) disruption of male orientation to female-proxy assessment traps was observed throughout the flight season, regardless of type and density of formulation applied, or total amount of pheromone used (Figs. 1, 3–6). In addition, when whole orchards were pheromone-treated in 2011, catches of *C. strobilella* were much lower as compared with catches in non-treated orchards (Fig. 3). The dramatic decrease in trap catch did, however, often not correspond to a subsequent reduction in cone damage, in this study measured by the frequency of infested cones and larval numbers in cones (Figs. 4–6). All experiments using rubber septa as disruption dispensers failed to reduce cone damage, and signs of damage suppression were only observed when SPLAT droplets were used at a relatively high density and total dose of active compound(s) (Figs. 5, 6). Whether this difference in success was due to the type of dispenser used or the dose of pheromone applied remains an open question. In addition, these optimisations resulted in a reduction in crop damage only in half of the trials performed for each moth species (Figs. 5, 6), demonstrating the challenges involved in mating disruption of these cone feeders. Our results are thus similar to those obtained in other studies on lepidopteran pests, for which a drastic decrease in trap catch has not been indicative of successful population suppression or reduced damage on the crop (Kerns 2000; Saucke et al. 2014; Mori and Evenden 2015a).

In our initial experiments on *C. strobilella*, we used a similar experimental protocol (type of disruption dispenser, dispenser density, dose of pheromone, size of treated area) compared to those used in the mating disruption experiments on *C. youngana* performed in Canadian spruce seed orchards (Trudel et al. 2006). In that study, the reduction in trap catch in the pheromone-treated area versus control area (87–98%) was generally less pronounced compared to our study, and catches in a treated area could be as high as 27 males per trap (Trudel et al. 2006). In spite of this lower level of communication disruption based on trap catches, a significant reduction in cone damage in the treated plot versus control plot was achieved for both years of the study (55 and 69%, respectively). These results on *C. youngana* are thus in contrast to ours on *C. strobilella*, where no reduction in cone damage was observed when using rubber disruption dispensers containing similar or even ten times higher doses of pheromone compared to the Canadian study. The different results obtained for these sister species of moths with very similar ecology are hard to explain, and there is a need for further studies to optimise pheromone-based mating disruption for *C. strobilella*.

Both moths in this study are known to fluctuate greatly in population size among sites within a given year as well as between years. We thus used, except in 2011, an approach applied in many other similar studies, i.e. using a pheromone treatment plot and a control plot in the same locality to enable reliable comparisons of trap catch and crop damage between such plots while standardising for the local population size of the target species. When using such experimental design, mated females may disperse from adjacent untreated areas into the pheromone-treated area, resulting in high levels of crop damage in spite of a true disruption effect on mating behaviour within the treated area. In 2011, when all six sites in southern Sweden had sufficient flowering to allow analysis of crop damage, we pheromone-treated the entire area of the smallest seed orchards (Gälltofta and Maglehem) and left the remaining sites untreated, but found no sign of reduced infestation of *C. strobilella* in treated versus untreated sites. Data obtained from such a study are obviously difficult to evaluate due to low replication and lack of information about local population densities of target species. Moreover, in the trials with *D. abietella*, parallel treatments with Bt in most locations in this year drastically reduced larval numbers in cones, making it impossible to evaluate the efficacy of the pheromone treatment. To directly assess whether the pheromone application reduces the frequency of mated females within a treated area, tethered moths or mating tables have to be used (Stelinski et al. 2005; Svensson et al. 2001), but such experiments were not performed in the present study.

Performing mating disruption studies on pests in Swedish spruce seed orchards is a challenge because few sites are available for experiments each year due to irregular and unpredictable flowering, and the low replication limits the evaluation of data from trapping trials and cone analyses. In addition, the populations of the target species at a given site can fluctuate heavily between years, making it difficult to select comparable sites for experiments and to compare datasets from different years. Similar problems occur when evaluating different treatments, e.g. mating disruption, against indoor pests as discussed in detail by Sieminska et al. (2009). However, indoor pests usually show smaller population fluctuations compared to the cone feeders analysed in this study, which rely on spruce flowering, and therefore, various kind of temporal comparisons can be done (Ryne et al. 2006, 2007; Sieminska et al. 2009). On the other hand, the damage caused by many indoor pests is more “diffuse” and various indirect estimates of damage or populations have to be used, as summarised in Anderbrant et al. (2009). In this study, we used all available sites for experiments over several seasons to study the potential of pheromone-based control of two spruce pests, and by accumulating experience from trials using different methods and performed in different locations, we think we have been able to show a reliable mating disruption effect on these pests as was the case for the indoor pests.

The results from the electrophysiological recordings using a portable device indicated elevated relative concentrations of pheromone in the air in the pheromone-treated plot. A small but significant increase in antennal response for *C. strobilella* was observed for recordings at 4 m height in the treatment area versus recordings in the control area when disruption dispensers were present, whereas such difference disappeared after the dispensers had been removed (Fig. 2), and EAG data thus correlated well with trapping data, showing a recovery in catches after dispenser removal (Fig. 1). Recordings with portable EAG equipment the way we used it cannot determine absolute pheromone concentrations and are influenced by the presence of other environmental volatiles. However, such recordings can still provide important information regarding the spatial and temporal distribution of active compounds to refine the strategy of mating disruption in small size habitats such as fruit orchards and seed orchards (Karg and Sauer 1995; Milli et al. 1997).

Studies on lepidopteran seed predators in other crop systems have demonstrated the challenges in achieving efficient mating disruption. The red clover casebearer moth, *Coleophora deauratella*, is an invasive pest in clover seed production in North America (Evenden et al. 2010), for which several techniques for mating disruption have recently been evaluated. When using reservoir-type rope dispensers, catches were reduced by > 99% but no measures of crop damage were taken (Mori and Evenden 2014). In a subsequent study using puffers for high release of pheromone, attraction of males to synthetic lures was suppressed by 61% in 0.25 ha plots and by 94% in 5 ha plots, but crop infestation was not significantly reduced (Mori and Evenden 2015a). In contrast, laminate flake pheromone dispensers provide significant suppression of trap catches (72–94%), which was correlated with reduction in larval numbers (Mori and Evenden 2015b). These studies show the importance of testing different mating disruption strategies to achieve successful control of a pest.

In conclusion, our study illustrates the challenges involved in pheromone-based population control of *C. strobilella* and *D. abietella* in spruce seed orchards. Although trap catches were suppressed to almost zero in most experiments, a corresponding decrease in crop damage was only observed when SPLAT formulations were applied at high density and at a high dose of active compounds, and only in half of the trials per species. Immigration of mated females was likely a factor causing mating disruption failure in this study. To limit such an effect in future attempts to control these moths, the entire orchard should be treated. Given the fairly large areas of spruce seed orchards, and the large size of the trees in these orchards, application of dispensers at crown level by hand will be very time-consuming and labour-intensive, and aerial application of pheromone, which has been used to treat other moth pests, e.g. *Epiphyas postvittana* (Brockerhoff et al. 2012), may instead be used. The development of pheromone-based methods for monitoring and control of pest insects agrees well with the EU directive to implement integrated pest management (IPM) principles to reduce pesticide use in crop systems (European Commission 2009). Further optimisation of the mating disruption method is, however, needed to achieve efficient control of *C. strobilella* and *D. abietella* and increase the seed yields in European spruce seed orchards.

## Author contributions

GPS, OR, CL, and OA designed the research. GPS, HLW, EVJ, OA, and OR conducted the experiments. IL and JMC contributed pheromone components. GPS and OR analysed the data. GPS wrote the manuscript. All authors read and approved the manuscript.

### Electronic supplementary material

Below is the link to the electronic supplementary material.

Supplementary material 1 (JPEG 388 kb)

Supplementary material 2 (DOCX 76 kb)

Supplementary material 3 (DOCX 47 kb)

Supplementary material 4 (DOCX 57 kb)

## Appendix 1: Synthesis of (8*E*,10*Z*)-dodecadienyl acetate

Scheme: Reagents and conditions: (a) *t*-BuOK, THF, NMP, −78 °C, 1 h; (b) Cl(O)P(OEt)_2_, −78 °C to 20 °C, 2 h, 89%; (c) 48% HBr, toluene, reflux, 48 h, 93%; (d) DHP, DCM, Amberlyst-15, 20 °C, 6 h, 95%; (e) Mg, THF, reflux, 2 h; (f) 1% Fe(acac)_3_, THF, 20 °C, 30 min, 73%; (g) MeOH, Amberlyst-15, 55 °C, 5 h, 90%; (h) Ac_2_O, Py, 0–20 °C, 5 h, 93%.

Abbreviations: *t*-BuOK—potassium *tert*-butoxide; THF—tetrahydrofuran; NMP—*N*-methylpyrrolidine; Cl(O)P(OEt)_2_—diethyl chlorophosphate; HBr—hydrogen bromide; DHP—3,4-dihydro-2*H*-pyran; DCM—dichloromethane; Fe(acac)_3_—iron(III) acetylacetonate; MeOH—methanol; Ac_2_O—acetic anhydride; Py—pyridine.

According to the scheme, (*E,Z*)-dienol phosphate **3** was prepared via a one-pot procedure that involves stereoselective enolisation of α,β-ethylenic aldehyde **2** with *t*-BuOK in the presence of NMP followed by trapping the resulting dienolate with Cl(O)P(OEt)_2_ (Cahiez et al. 2008a). Phosphate **3** was obtained in 89% yield with isomers ratio *EZ*/*EE* = 92/8. Dienol phosphate was subjected to the iron-catalysed cross-coupling alkylation with the Grignard reagent **4** from THP-protected bromohydrin to yield THP-protected 8*E*,10*Z*-dodecadienol (Cahiez et al. 2008b). The desired 8*E*,10*Z*-dodecadienyl acetate **1**, which has isomeric purity up to 86.8% (other isomers: 0.5% *ZE*-, 10% *EE*-, 2.7% *ZZ*-), was obtained after deprotection and acetylation.

## Appendix 2: Synthesis of (3*Z*,6*Z*,9*Z*,12*Z*,15*Z*)-pentacosapentaene

Scheme: Reagents and conditions: (a) LiAlH_4_, ether, 0–20 °C, 12 h, 99%; (b) *p*-TsCl, cat. DMAP, Py, DCM, 0–20 °C, 12 h, 99%; (c) CH_3_(CH_2_)_4_MgBr, 6% Li_2_CuCl_4_, ether–THF, −50 to +5 °C, 10 h, 83%.

Abbreviations: LiAlH_4_—lithium aluminium hydride; ether—diethyl ether; *p*-TsCl—*para*-toluenesulfonyl chloride; DMAP—4-*N,N*-dimethylaminopyridine; Py—pyridine; DCM—dichloromethane; THF—tetrahydrofuran.

According to the scheme (Millar et al. 2005), eicosapentaenoic acid ethyl ester (**6**, KD Pharma) was reduced with LiAlH_4_ to afford the expected primary alcohol **7** which was then converted to its tosylate **8** by reaction with *p*-TsCl. Coupling of this tosylate with the Grignard reagent of 1-bromopentane using a copper catalyst (Fouquet and Schlosser 1974, Schlosser 1974) gave the desired pentaene **5**, which has isomeric purity 96–98%.
