# Supplementary figures and images for: Challenges of pheromone-based mating disruption of Cydia strobilella and Dioryctria abietella in spruce seed orchards
Source: J Pest Sci (2004). 2017 Nov 7;91(2):639–50. doi: 10.1007/s10340-017-0929-x (PMC5847141; doi:10.1007/s10340-017-0929-x)

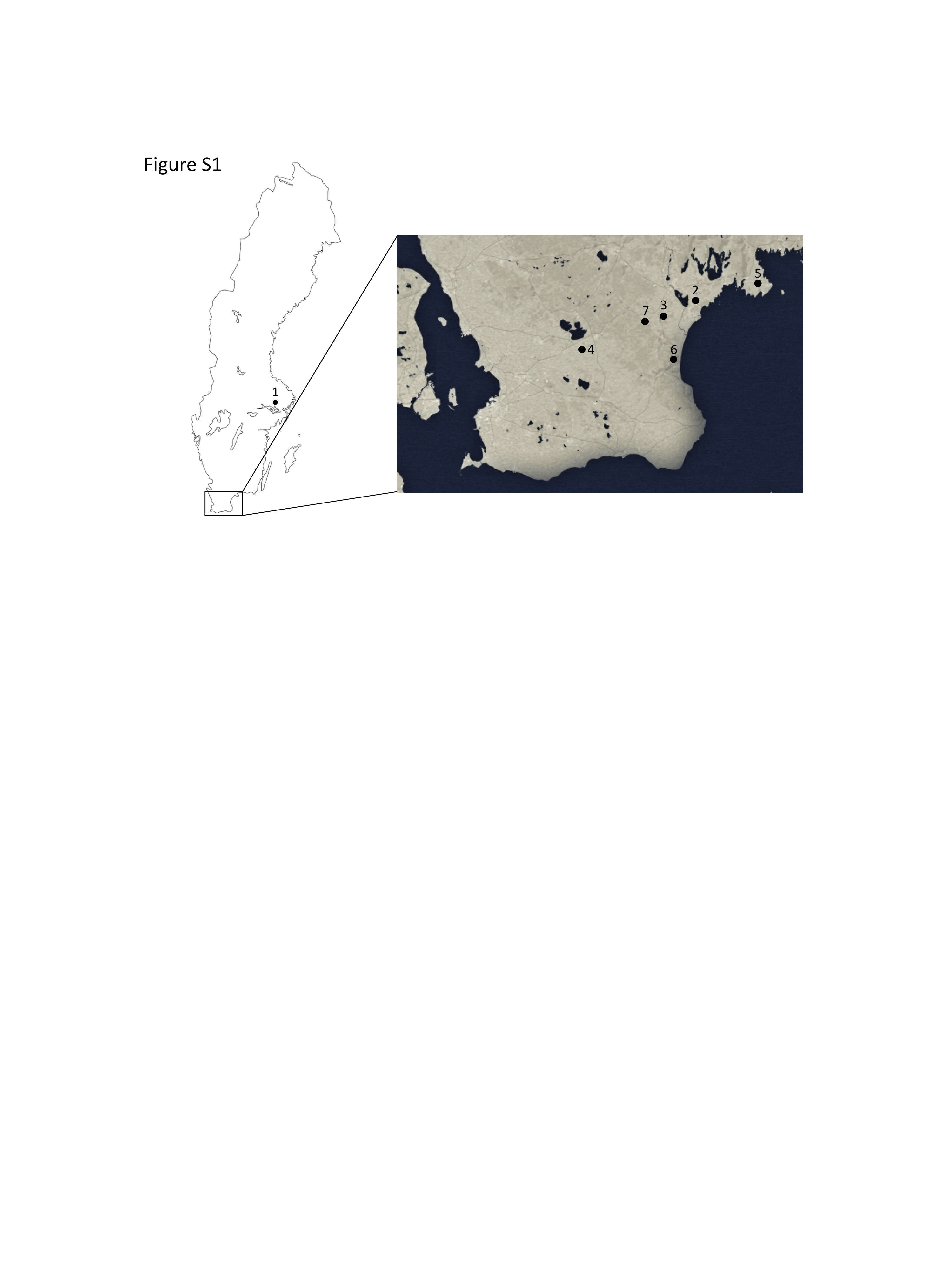

Supplement: Supplementary file 1 — Supplementary material 1 (JPEG 388 kb) [file 10340_2017_929_MOESM1_ESM.jpg]
