# Supplementary material for: Challenges of pheromone-based mating disruption of Cydia strobilella and Dioryctria abietella in spruce seed orchards
Source: J Pest Sci (2004). 2017 Nov 7;91(2):639–50. doi: 10.1007/s10340-017-0929-x (PMC5847141; doi:10.1007/s10340-017-0929-x)
Supplement: Supplementary file 2 — Supplementary material 2 (DOCX 76 kb) [file 10340_2017_929_MOESM2_ESM.docx]

**Table S1**: Origin, purity, and application of compounds used in different mating disruption (MD) experiments on *Cydia strobilella* and *Dioryctria abietella* during 2010, 2011, 2012 and 2015.

Compound Origin Chemical purity Isomeric purity Year MD dispenser/bait/EAG

*E*8,*E*10-12:OAc Bedoukian^1^ >98% >98% All years MD dispenser/bait

*E*8,*E*10-12:OAc Bedoukian^1^ >98% >98% 2011 MD dispenser

*E*8,*E*10-12:OAc IOB^2^ >95% >99% 2010, 2011 Bait/EAG

*E*8,*Z*10-12:OAc Synthesised >80% >88% All years MD dispenser/bait

*E*8,*Z*10-12:OAc Synthesised >57% >87% 2012, 2015 MD dispenser

*E*8,*Z*10-12:OAc Synthesised >40% >83% 2015 MD dispenser

*E*8,*Z*10-12:OAc Donation^3^ >93% >94% 2010 Bait/EAG

*Z*9,*E*11-14:OAc Bedoukian^1^ >97% >98% 2010, 2011, 2015 MD dispenser/bait

C25 pentaene Synthesised >96% >96% 2010, 2011, 2014 MD dispenser/bait

^1^Bedoukian Research Inc., Danbury, CT, USA.

^2^IOB, Wageningen, The Netherlands.

^3^Provided by Prof. M. Bengtsson (Swedish University of Agricultural Sciences, Alnarp, Sweden).
