# Supplementary material for: Challenges of pheromone-based mating disruption of Cydia strobilella and Dioryctria abietella in spruce seed orchards
Source: J Pest Sci (2004). 2017 Nov 7;91(2):639–50. doi: 10.1007/s10340-017-0929-x (PMC5847141; doi:10.1007/s10340-017-0929-x)
Supplement: Supplementary file 3 — Supplementary material 3 (DOCX 47 kb) [file 10340_2017_929_MOESM3_ESM.docx]

**Table S2**: Sites used for mating disruption experiments on *Cydia strobilella* and *Dioryctria abietella*, their geographic location and size.

Location Province Geographic coordinates Area (ha)

Ålbrunna Uppland 59°30'00''N, 17°32'00''E 25

Gälltofta Skåne 55°58'32''N, 14°19'25''E 6

Gringelstad Skåne 55°55'40"N 14°06'17"E 27

Högseröd Skåne 55°48'15''N, 13°34'45''E 8

Hosaby Blekinge 56°02'05''N, 14°41'39''E 13

Maglehem Skåne 55°46'09''N, 14°09'48''E 6

Maltesholm Skåne 55°54'49"N 13°59'33"E 8
