# Supplementary material for: Challenges of pheromone-based mating disruption of Cydia strobilella and Dioryctria abietella in spruce seed orchards
Source: J Pest Sci (2004). 2017 Nov 7;91(2):639–50. doi: 10.1007/s10340-017-0929-x (PMC5847141; doi:10.1007/s10340-017-0929-x)
Supplement: Supplementary file 4 — Supplementary material 4 (DOCX 57 kb) [file 10340_2017_929_MOESM4_ESM.docx]

**Table S3:** Main characteristics of experiments on *Cydia strobilella* (C.s.) and *Dioryctria abietella* (D.a.) conducted 2010, 2011, 2012 and 2015.

Year Species Site Area treated Dispenser Dose/ha Point sources/ha Dispenser (ha) type (g) height (m)

2010 C.s. Maltesholm 1 Rubber septum 0.128 64 2 or 4

C.s. Ålbrunna 1 Rubber septum 0.128 64 2 or 4

2011 C.s./D.a. Gälltofta 6 Rubber septum 0.128/6.4 64 2

Gringelstad ctrl

Högseröd ctrl

Hosaby ctrl

Maglehem 6 Rubber septum 0.128/6.4 64 2

Maltesholm ctrl

2012 C.s. Ålbrunna 1 Rubber septum 1.28 64 4-5

Hosaby 1 Rubber septum 1.28 64 4-5

Maglehem 1 Rubber septum 1.28 64 4-5

2015 C.s. Hosaby 2 SPLAT 6.4 320 4-5

Maltesholm 2 SPLAT 6.4 320 4-5

D.a. Gringelstad 2 SPLAT 50 320 4-5

Maglehem 2 SPLAT 50 320 4-5
